# KRAS mutation promotes the colonization of Fusobacterium nucleatum in colorectal cancer by down‐regulating SERTAD4

**DOI:** 10.1111/jcmm.70182

**Published:** 2024-10-27

**Authors:** Yizhen Chen, Yuanyuan Zheng, Shaolin Liu

**Affiliations:** ^1^ Department of Geriatric Medicine, Fujian Key Laboratory of Geriatrics Diseases, Fujian Provincial Center for Geriatrics, Fujian Provincial Hospital Fuzhou University Affiliated Provincial Hospital, School of Medicine, Fuzhou University Fuzhou Fujian China; ^2^ Shengli Clinical Medical College of Fujian Medical University Fuzhou Fujian China

**Keywords:** colorectal cancer, Fusobacterium nucleatum, KRAS, prognosis

## Abstract

This study explores and verifies potential molecular targets through which KRAS mutations regulate the colonization of Fusobacterium nucleatum (FN) in colorectal cancer (CRC). This study combined multiple bioinformatics methods and biological assays. Through The Cancer Genome Atlas, Gene Expression Omnibus, Human Protein Atlas, immunohistochemistry, and co‐culture assays, we further confirmed the differential expression of SERTAD4 in CRC. We delved deeper into examining how expression of SERTAD4 is linked with immune cell infiltration and the enrichment of potential pathways. Lastly, through bacterial phenotypic assays, we validated the function of SERTAD4. As a molecule associated with KRAS mutations and FN infection, the expression levels of SERTAD4 were downregulated in CRC. The diagnostic efficacy of SERTAD4 for CRC is not inferior to that of CEA. Low expression of SERTAD4 is associated with poorer overall survival in CRC. Correlation analysis found that increased expression of SERTAD4 is associated with various immune cell infiltrations and immune checkpoint genes. Finally, bacterial adhesion and invasion assays verify that SERTAD4 inhibits the adhesion and invasion abilities of FN in CRC. This study demonstrates that SERTAD4 exerts a protective role in CRC by inhibiting the colonization of FN.

## 1 INTRODUCTION

Globally, the incidence and mortality rates of colorectal cancer (CRC) remain high.^1^ KRAS mutations lead to the sustained activation of carcinogenic signals and downstream oncogenes.^2^ Numerous clinical studies have found that, compared to CRC patients with KRAS wild‐type (WT), those with KRAS mutation (MUT) have a poorer prognosis.^3^, ^4^, ^5^ Consequently, there is a pressing necessity to discover new and dependable targets to enhance the prognosis for CRC patients harbouring KRAS mutations.

The human gut is rich in microbes, and dysbiosis of the gut microbiota has been identified as a significant factor influencing CRC.^6^ Notably, Fusobacterium nucleatum (FN) is an anaerobic opportunistic pathogen that is highly enriched in the faeces and tumour tissues of CRC patients.^7^, ^8^ Elevated the abundance of FN have been linked to decreased overall survival (OS) rates in CRC.^9^, ^10^, ^11^, ^12^, ^13^, ^14^ FN contributes to the development and progression of CRC by stimulating tumour proliferation, affecting the tumour microenvironment (TME), and inducing chemotherapy resistance.^15^, ^16^ These emphasize the importance of targeting FN in the prevention and treatment of CRC. As understanding of the carcinogenic mechanisms of gut microbiota, how to target FN to improve the prognosis of CRC remains unclear.

Manipulating the gut microbiome to enhance the treatment response of CRC is a new perspective.^17^, ^18^ Developing therapeutic methods targeting specific carcinogenic bacteria has become a novel treatment approach. Several studies hint that in CRC patients, the abundance of FN is related to tumour size and KRAS mutations.^19^, ^20^ Therefore, this study aims to integrate various bioinformatics methods to explore potential molecular targets that KRAS mutations regulate for colonization of FN in CRC. We systematically screened gene sets associated with KRAS mutations and FN infection by Gene Expression Omnibus (GEO). Through CRC samples and co‐culture assays, we found that both KRAS mutation and FN infection suppress the expression of SERTAD4. Consequently, we investigated the expression profile of SERTAD4 as a molecule related to KRAS mutation and FN infection in CRC, and its association with clinical outcomes.

We examined the variance in expression of SERTAD4 within CRC using data from The Cancer Genome Atlas (TCGA) database and validated SERTAD4 in the GEO database and CRC samples. The protein levels of SERTAD4 were detected using the Human Protein Atlas (HPA) and immunohistochemistry (IHC). We assessed the diagnostic and prognostic potential of SERTAD4 in CRC using both GEO and TCGA datasets, later validating our findings with CRC patient samples. Our research focused on exploring the relationship between expression levels of SERTAD4, immune cell infiltration and the expression of immune checkpoint genes. Through pathway enrichment analysis, we uncovered the potential importance of SERTAD4 in the context of CRC. Finally, the function of SERTAD4 was further verified through bacterial phenotypic and cellular phenotypic assays.

## 2 MATERIALS AND METHODS

### 2.1 Colorectal cancer samples and data collection

**FIGURE 1:**
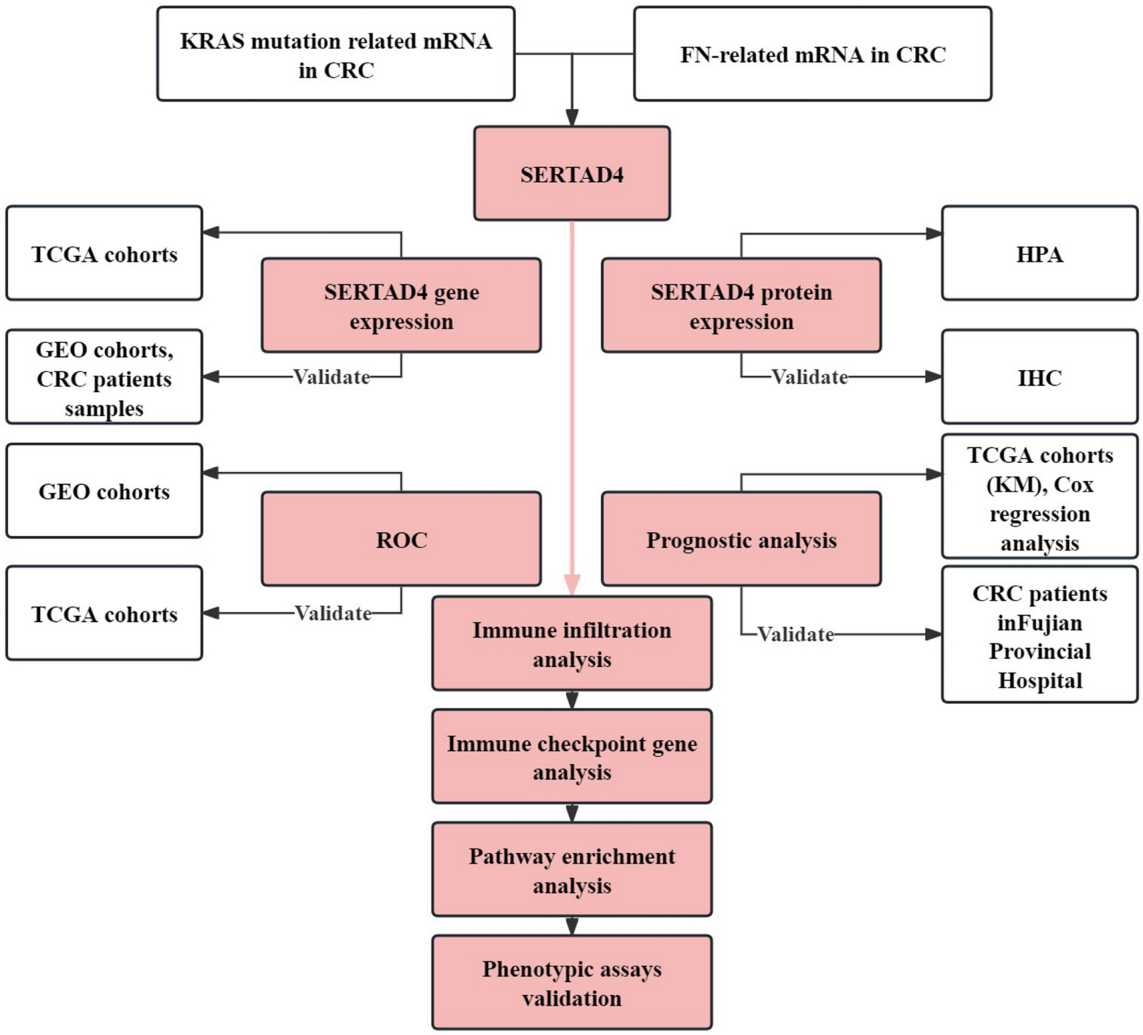
Study design and related assays flowchart. This illustrates the design and related assays of this study.

Related datasets were obtained from the GEO database.^21^ We detected the differential mRNA levels of SERTAD4 between CRC and normal colonic epithelial tissues. The mRNA level differences of SERTAD4 between KRAS MUT CRC with and KRAS WT were analysed through CRC samples. Furthermore, analysis was conducted on the data downloaded from TCGA, which includes standardized expression data and related clinical information.^22^ The protein levels of SERTAD4 were primarily analysed through the HPA database (https://www.proteinatlas.org/) and further validated by IHC.^23^ We collected tumour tissues from CRC patients at Fujian Provincial Hospital (see Table S1 for details). The design of this study is shown in Figure 1.

### 2.2 Cell culture

Human CRC cell lines (RKO, HCT116, SW48, LOVO, SW620, HT29, and SW480) were purchased from China Haisen Biotechnology Co., Ltd. The above cells were cultured in the medium.

### 2.3 Cell Counting Kit‐8 proliferation assay

CRC cells in log phase were digested with trypsin and then centrifuged. The cells were resuspended in medium and seeded in the 96‐well plate. Five replicate wells were set for each group. Ten microliter of the Cell Counting Kit‐8 (CCK‐8) solution was dispensed into every well. The optical density (OD) at 450 nm was measured using a microplate reader.

### 2.4 Colony‐formation assay

CRC cells in log phase were digested with trypsin, centrifuged and resuspended. Cells were allocated into a six‐well plate at a rate of 500 cells per well. Following this, the six‐well plate was transferred to an incubator where it was cultured for a duration of 7–10 days. The experiment was concluded once discernible colonies were observed in the culture dishes. After fixing the cells, crystal violet staining was applied. Photographs were taken for counting, with three replicate wells set up for each group.

### 2.5 Fusobacterium nucleatum culture, co‐culture and bacterial phenotype assays

The resuscitation and culture of FN were strictly carried out in accordance with the instructions.^24^ The planktonic growth and density of FN were monitored by measuring the OD at 600 nm. All assays ensured that both bacteria and cells were in the logarithmic growth phase. The bacterial phenotype assays in this study included bacterial adhesion and invasion assays. The bacterial adhesion assay was conducted as follows: bacteria were co‐cultured with cells (multiplicity of infection (MOI) of 1:10).^25^ After incubation under anaerobic conditions at 37°C for 4–6 h, the culture was washed three times with 1× sterile PBS to remove non‐adherent bacteria. Cells bearing attached bacteria were treated with trypsin at 37°C for 3 min. To halt the reaction, culture medium enriched with serum was added. Subsequently, the bacterial mixture was evenly spread on blood agar plates using the dilution plating technique and incubated anaerobically at 37°C for 48 h. The bacterial invasion assay differed slightly from the steps described above. The co‐culture period of bacterial and cell was 12 h. Additionally, after co‐culturing, a culture medium containing cefoxitin (100 μg/mL) was used for 5 min to kill the bacteria that did not invade the cells. The colony forming units on each plate represented the adhesion and invasion capabilities of the bacteria. Each group was repeated three times. Colonies numbering between 30 and 500 and evenly distributed were considered reasonable.

### 2.6 Reverse transcription quantitative PCR

PCR amplification and cycle threshold (CT) value measurement were performed using the Roche Light Cycler 480 system. The reagents required for reverse transcription and amplification were all obtained from Fujian Horay Biotechnology Co., Ltd. The primer sequences for SERTAD4 are as follows: Forward: 5′‐AGGAAGTATGTGGAAGAAGAGGAT‐3′, Reverse: 5′‐TTGTTGCGAAGTTGATGTCTCT‐3′. GAPDH was used as an internal reference gene. The primer sequences for FN are as follows: Forward: 5′‐CAACCATTACTTTA.

ACTCTACCATGTTCA 3′, Reverse: 5′‐GTTGACTTTACAGAAGGAGATTATGTAAAAATC3′. The relative expression level of SERTAD4 was calculated using the 2 −ΔΔCT method.

### 2.7 RNA interference

Seed the cells in a 6‐well plate. When the cell density reaches 65%–75%, transfect with small interfering RNAs (siRNA). Add 150 μL of diluted Lipofectamine 3000–150 μL of diluted plasmid. Then mix well to encapsulate the plasmid with liposomes. Add 300 μL of the liposome‐plasmid DNA complex to the cells and gently mix. After incubating for 12 h, change the medium of the transfected cells.

### 2.8 Immunohistochemistry and western blotting

Fix the tissue samples onto slides, then proceed with dehydration, paraffin impregnation and paraffin embedding. Place the paraffin sections in an oven at 60°C (overnight). Then, carry out a series of dewaxing and hydration steps. To enhance the binding of antibodies to antigens, perform antigen retrieval. Add Anti‐SERTAD4 antibody (ab121997, Abcam, UK) to the samples and incubate overnight at 4°C. Incubate with secondary antibodies. The colour development reaction is performed with diaminobenzidine. Observe and take photos under a microscope. All results of IHC were independently evaluated by two observers using the staining index (SI). Western blotting was implemented as described in references.^26^ Anti‐SERTAD4 antibody was purchased from Santa (YS‐19662R, USA).

### 2.9 Diagnostic and prognostic value of SERTAD4 in colorectal cancer

Use R (version 4.2.1) with pROC [1.18.0], ggplot2 [3.3.6] to calculate the area under the curve (AUC) of the receiver operating characteristic (ROC) curves. We analyse the survival data obtained from TCGA using survival [3.3.1], survminer [0.4.9] and ggplot2 [3.3.6].

### 2.10 Analysis of immune cell infiltration

Using the ssGSEA algorithm in the R package‐GSVA [1.46.0], we calculated the corresponding immune infiltration in the TCGA database using markers for 24 types of immune cells provided by previous studies.^27^, ^28^

### 2.11 Pathway enrichment analysis

Analyse and screen out the differentially expressed genes (DEGs) of SERTAD4. Use clusterProfiler [4.4.4] to perform functional annotation and pathway enrichment analysis on the DEGs. Employ ggplot2 [3.3.6] to visualize the results.

### 2.12 Statistical analysis

This study utilized R software, GraphPad Prism (9.5.1), and online analysis websites for data analysis. Comparisons between groups were made using the Wilcoxon rank‐sum test or Student's *t* test. Differences in survival were evaluated using kaplan–meier (KM). A *p*‐value of <0.05 was considered statistically significant.

## 3 RESULTS

### 3.1 Identification of mRNA related to KRAS mutation and Fusobacterium nucleatum infection in colorectal cancer

**FIGURE 2:**
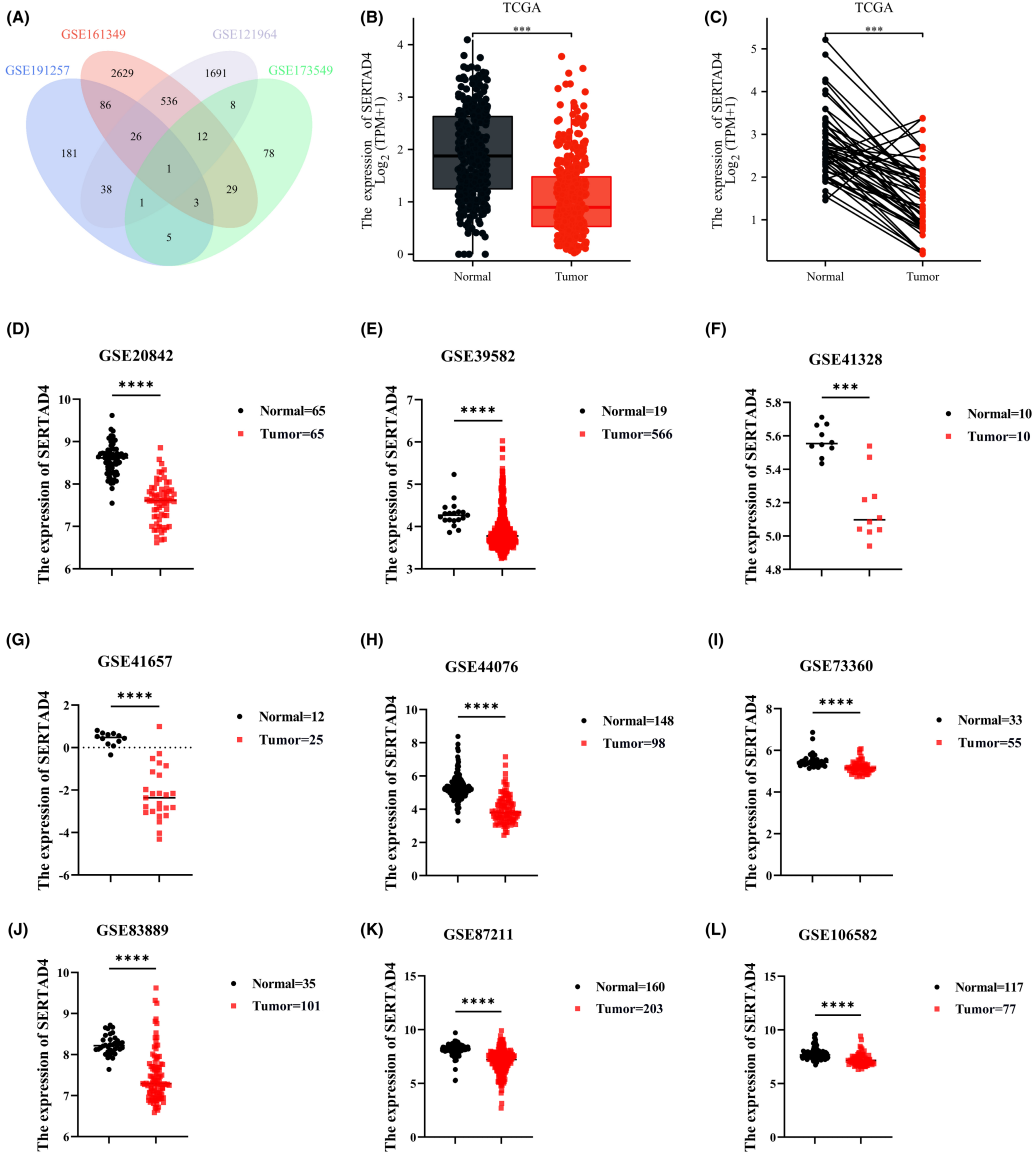
The RNA levels of SERTAD4 in CRC. (A) SERTAD4 was identified by intersecting Venn diagrams after searching the GEO dataset. (B) Analysing the RNA levels of SERTAD4 in CRC tissues and normal intestinal epithelial tissues based on TCGA. (C) Analysing the RNA levels of SERTAD4 in CRC tissues and matched normal intestinal epithelial tissues based on TCGA. Analysing the RNA levels of SERTAD4 in CRC tissues and normal intestinal epithelial tissues based on GSE20842 (D), GSE39582 (E), GSE41328 (F), GSE41657 (G), GSE44076 (H), GSE73360 (I), GSE83889 (J), GSE87211 (K), and GSE106582 (L). (*p* > 0.05, ns, nonsignificant; *p* < 0.001 ***; *p* < 0.0001 ****; analyses were performed using Student' s *t* test or Wilcoxon rank‐sum test, respectively).

By intersecting mRNA associated with KRAS mutations in CRC (GSE121964 and GSE161349) and FN infection (GSE173549 and GSE191257), we identified a unique mRNA that met the above criteria: SERTAD4 (Figure 2A). We first analysed SERTAD4 in CRC and normal intestinal epithelial tissues in the TCGA database, and the findings indicated that SERTAD4 expression was notably greater in normal tissues compared to CRC tissues (Figure 2B, *p* < 0.05). Furthermore, additional analysis in the TCGA database of CRC and paired normal tissues also demonstrated that SERTAD4 expression was notably greater in normal tissues (Figure 2C, *p* < 0.05). To further validate the higher expression of SERTAD4 in normal tissues, we searched for datasets with normal tissue and CRC tissue in the GEO database. The findings revealed that across multiple datasets, SERTAD4 expression was significantly elevated in normal intestinal epithelial tissues in contrast to CRC tissues (GSE20842, GSE39582, GSE41328, GSE41657, GSE44076, GSE73360, GSE83889, GSE87211 and GSE106582; Figure 2D–L, *p* < 0.05).

Aside from its expression in CRC, we were intrigued to discover if SERTAD4 exhibited variable expression levels across other cancer types. Analysis using TCGA data revealed that SERTAD4 expression was upregulated in 12 cancer types and downregulated in 7 cancer types (Figure S1A, all *p* < 0.05). We further analysed the difference between paired samples in pan‐cancer, and the results showed differences in paired samples in 12 types of cancer (Figure S1B, all *p* < 0.05). These observations indicate that SERTAD4 is not only crucial in CRC but also plays a significant role in various other cancers. In summary, mRNA level of SERTAD4 is significantly reduced in CRC.

**FIGURE 3:**
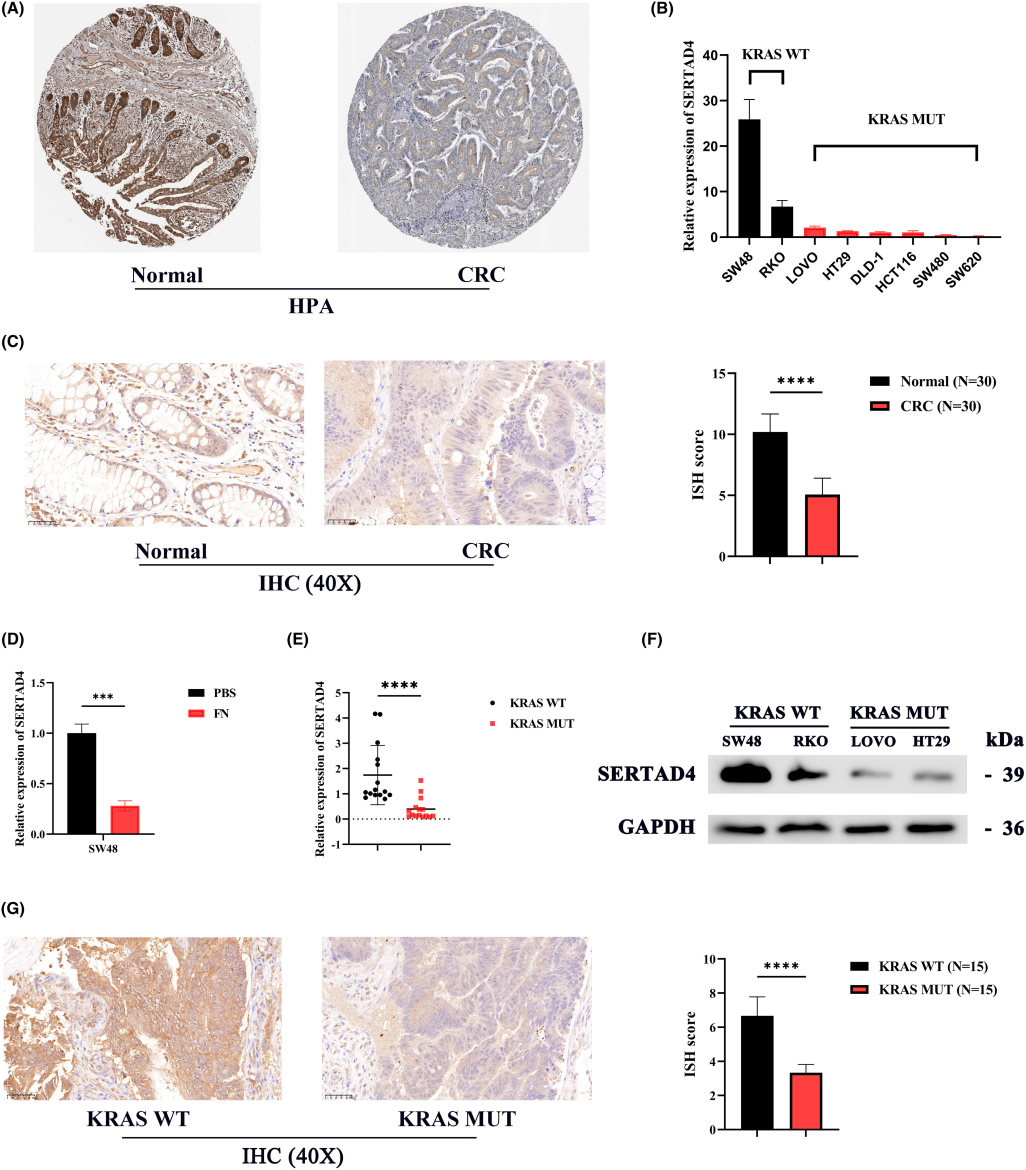
The protein levels of SERTAD4 in CRC and association with KRAS and FN. (A) Detected the protein expression of SERTAD4 in CRC tissues and normal tissues based on the HPA database. (B) Detected the expression level of SERTAD4 in 6 KRAS MUT and 2 KRAS WT CRC cell lines using RT‐qPCR. (C) Validated the protein expression of SERTAD4 in CRC tissues and normal tissues using IHC based on patient samples. (D) Detected the expression of SERTAD4 after co‐culturing cells with FN or PBS (control) using RT‐qPCR. (E) Detected the expression level of SERTAD4 in tumours from 15 KRAS MUT and 15 KRAS WT CRC patients using RT‐qPCR. (F) Detected the protein expression of SERTAD4 in 2 KRAS MUT and 2 KRAS WT CRC cell lines using WB. (G) Validated the protein expression of SERTAD4 in KRAS WT and KRAS MUT CRC patients using IHC based on patient samples. (*p* > 0.05, ns, nonsignificant; *p* < 0.001 ***; *p* < 0.0001 ****; analyses were performed using Student's *t* test or Wilcoxon rank‐sum test, respectively).

In addition to mRNA levels, we discovered through the HPA database that the protein expression of SERTAD4 is also significantly lower in CRC compared to normal tissues (Figure 3A). This result was validated by IHC in patient samples from Fujian Provincial hospital (Figure 3C). Having established that RNA and the protein levels of SERTAD4 are notably reduced in CRC tissues compared to normal intestinal epithelial tissues, we needed to further validate the correlation of SERTAD4 with KRAS mutations and FN infection in CRC. First, through reverse transcription quantitative PCR (RT‐qPCR), we discovered a reduction in the expression of SERTAD4 in CRC cell lines harbouring KRAS mutations (Figure 3B). Beyond studies in CRC cell lines, we conducted RT‐qPCR on 30 collected CRC tissues and discovered that SERTAD4 expression was lower in CRC tissues harbouring KRAS mutations compared to those with KRAS WT (Figure 3E, *p* < 0.05). Moreover, after 12 h of infection with FN (MOI 1:10) in CRC cell lines, we found that FN could also inhibit the expression of SERTAD4 (Figure 3D, *p* < 0.05). We finally confirmed through WB and IHC that SERTAD4 expression was lower in CRC with KRAS mutations than KRAS WT (Figure 3F,G). We further verified the correlation between the abundance of FN and SERTAD4 expression in clinical samples and found that the abundance of FN was negatively correlated with the expression of SERTAD4 (Figure S1C, *p* = 0.001). For the reliability of the study, we further confirmed the association of KRAS mutations with FN in CRC by qPCR. Figure S1D confirms that KRAS mutations are positively associated with high abundance of FN. In conclusion, compared to normal tissues, SERTAD4 expression is reduced in CRC tissues. Moreover, both KRAS mutations and FN infection suppress the expression of SERTAD4. In CRC, SERTAD4 is associated with KRAS mutations and FN infection.

### 3.2 SERTAD4 is a new diagnostic and prognostic biomarker in colorectal cancer

**FIGURE 4:**
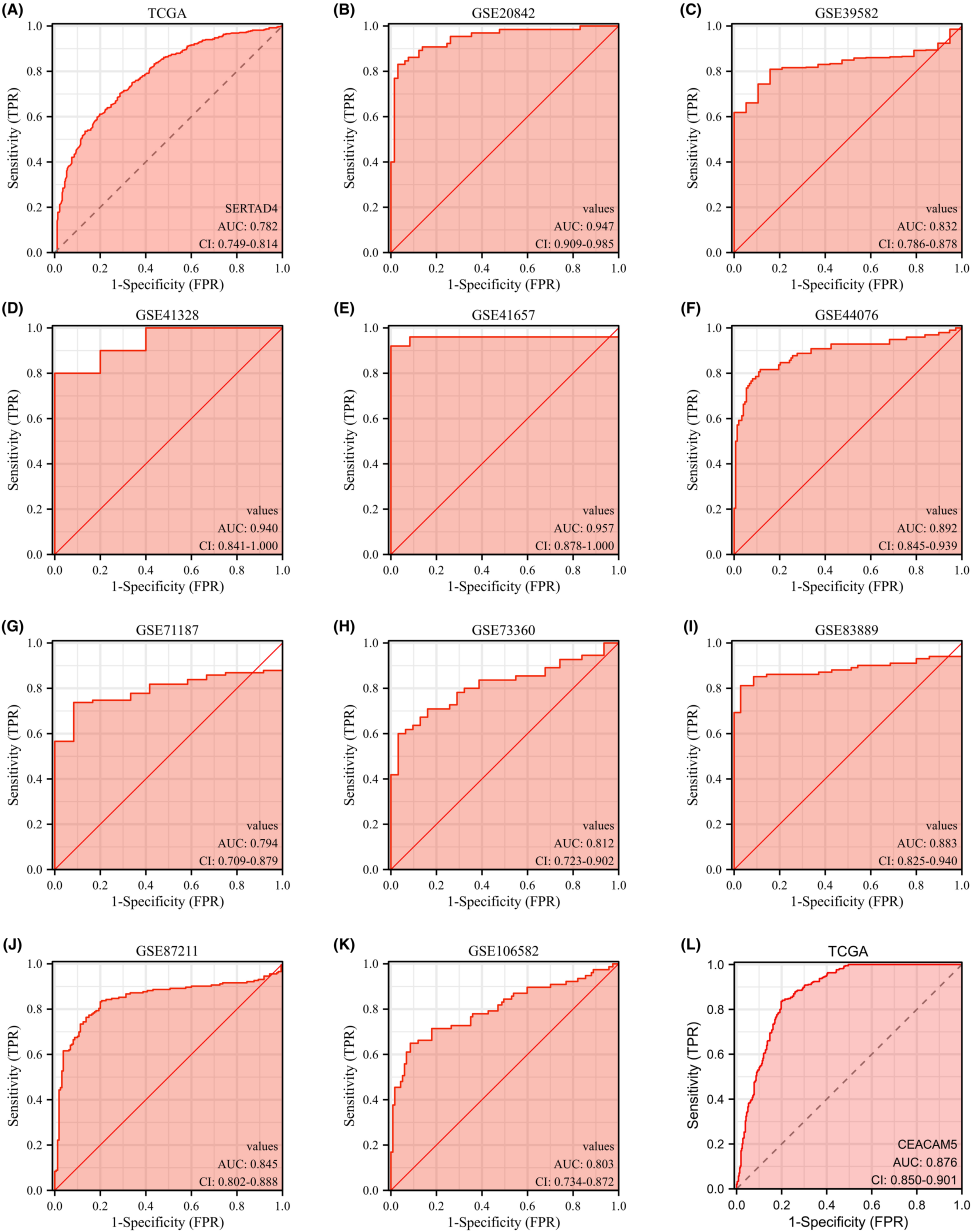
The diagnostic potential of SERTAD4 for CRC. (A) Based on TCGA, the diagnostic capability of SERTAD4 for distinguishing CRC patients from normal individuals. Based on GSE20842 (B), GSE39582 (C), GSE41328 (D), GSE41657 (E), GSE44076 (F), GSE71187 (G), GSE73360 (H), GSE83889 (I), GSE87211 (J), GSE106582 (K), the diagnostic capability of SERTAD4 for distinguishing CRC patients from normal individuals. (L) Based on TCGA, the diagnostic capability of CEA for distinguishing CRC patients from normal individuals.

Based on the differential expression of SERTAD4 in CRC, we sought to further explore its potential as a diagnostic tool for CRC. Retrieving patient data from TCGA‐GTEx (Normal = 359 vs. Tumour = 383), we found that SERTAD4 can distinguish CRC well (Figure 4A, AUC = 0.782). To further validate the diagnostic capability of SERTAD4 in CRC, we retrieved several classic CRC datasets from GEO. The results confirmed that SERTAD4 could effectively differentiate normal tissue from CRC and is a new molecule for diagnosis of CRC (Figure 4B–K, AUCs are 0.947, 0.832, 0.940, 0.957, 0.892, 0.794, 0.812, 0.883, 0.845 and 0.803, respectively). Moreover, analysing CEA, a classic tumour marker of CRC,^29^ we also evaluated its capability to diagnose CRC (Figure 4L, AUC = 0.876). It is evident that SERTAD4 has a diagnostic ability for CRC similar to that of CEA. These findings support SERTAD4 as a potential biomarker for CRC diagnosis.

**FIGURE 5:**
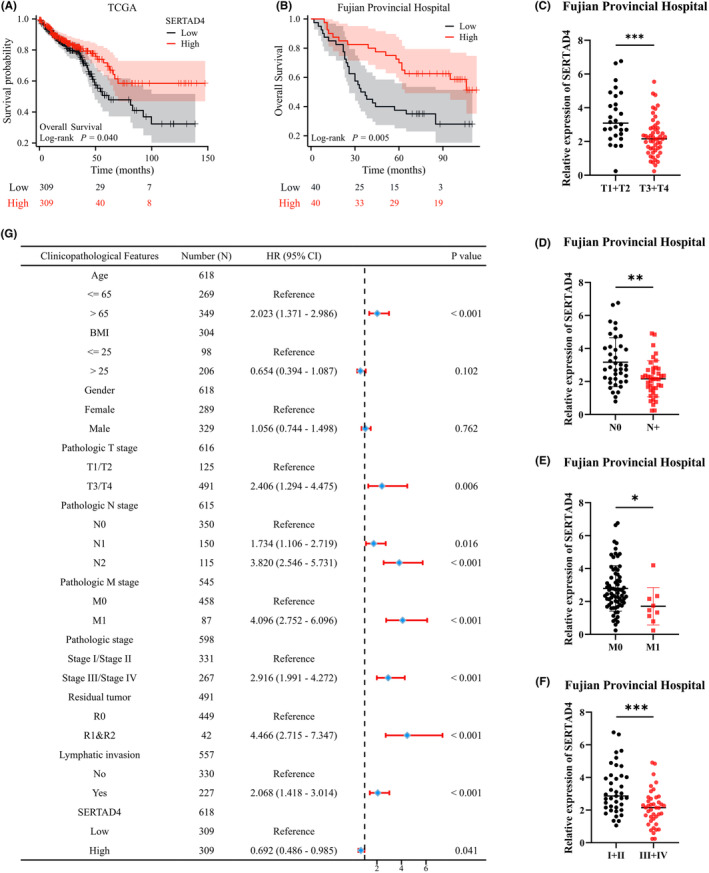
Low expression of SERTAD4 indicates poor prognosis in CRC patients. (A) Based on TCGA, KM survival curve analysis of the impact of SERTAD4 expression on OS in CRC patients. (B) Based on data from Fujian Provincial Hospital patients, KM survival curve analysis of the impact of SERTAD4 expression on OS in CRC patients. (C–F) The expression difference of SERTAD4 among CRC patients in different T, N, M, and TNM stages, respectively (*n* = 80 CRC patients). (G) A forest plot showing Cox regression analysis of the impact of SERTAD4 expression level on OS in CRC. (*p* > 0.05, ns, nonsignificant; *p* < 0.05 *; *p* < 0.01 **; *p* < 0.001 ***; analyses were performed using Student's *t* test or Wilcoxon rank‐sum test, respectively).

Furthermore, whether SERTAD4 can predict the prognosis of CRC is of interest to us. We first plotted the KM curves using TCGA data and then validated them with patients from Fujian Provincial Hospital. The TCGA results suggest that SERTAD4 expression is associated with better OS (Figure 5A, *p* = 0.040), which was validated by patients from Fujian Provincial Hospital (Figure 5B, *p* = 0.005). Furthermore, we assessed the association between SERTAD4 expression and clinical staging in CRC. The results demonstrate that a lower expression level of SERTAD4 is associated with advanced stages of T, N, M and TNM staging (Figure 5C–F,P<0.05). The findings strongly suggests that SERTAD4 could be linked to the proliferation, metastasis, and invasion of CRC, which certainly requires further assay validation. We delved deeper into TCGA data and developed a forest plot for OS using cox regression analysis, which further strengthened the notion of SERTAD4 as an independent protective factor in CRC (Figure 5G, HR = 0.692, *p* = 0.041). Moreover, we also analysed disease‐specific survival (DSS). After eliminating non‐cancer death patients, the protective effect of SERTAD4 on CRC was further enhanced (Figure S2A, HR = 0.648, *p* = 0.06). The analyses imply that the abnormal expression levels of SERTAD4 may have a significant association with the prognosis of patients with CRC. And, of course, how SERTAD4 participates in the progression of CRC needs further verification.

### 3.3 Immunological Research of SERTAD4 in colorectal cancer

**FIGURE 6:**
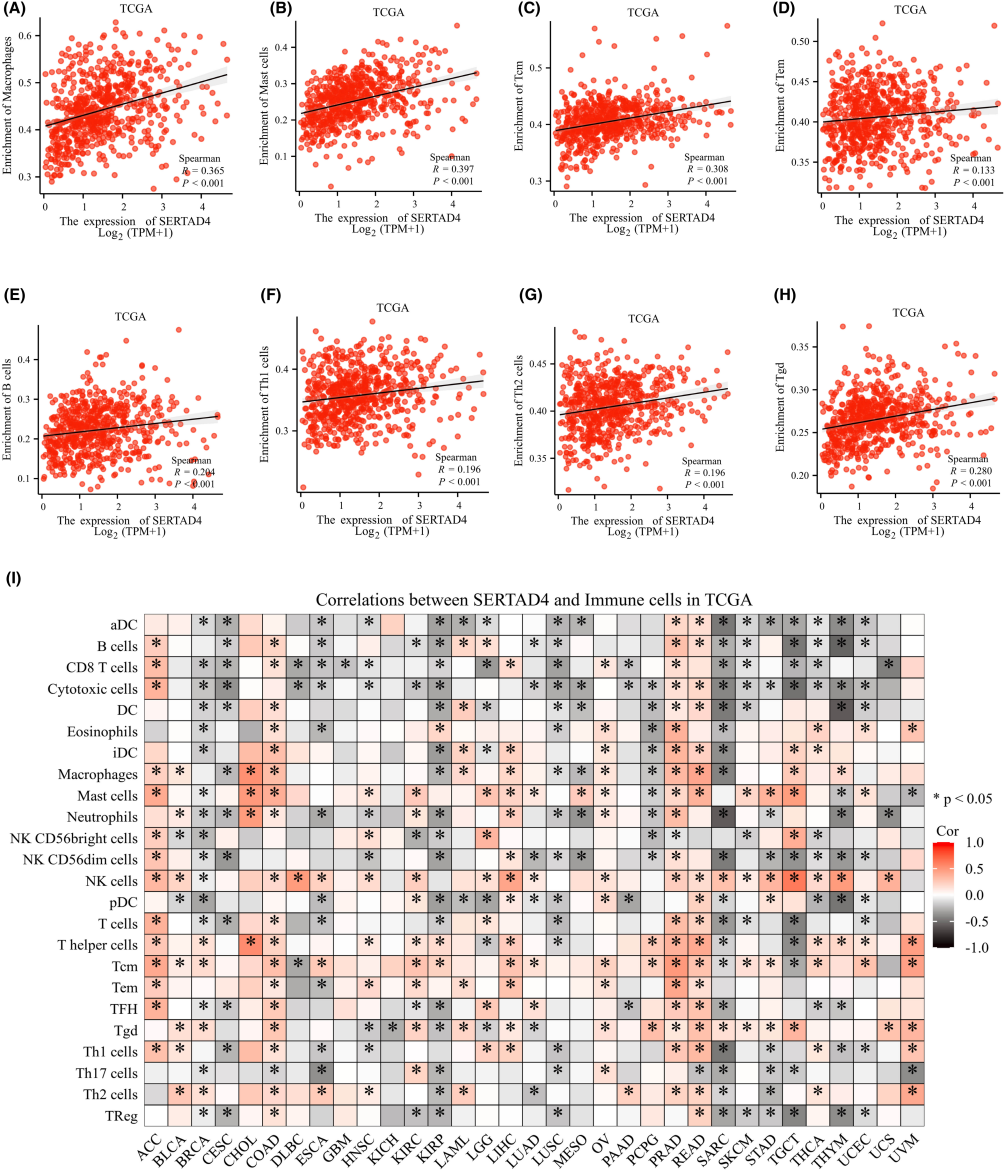
The relationship between expression levels of SERTAD4 and immune cell infiltration. The correlation of expression levels of SERTAD4 with macrophages (A), mast cell (B), tcm (C), tem (D), b cell (E), th1 cell (F), th2 cell (G), tgd (H). (I) Heatmap showing the relationship between expression levels of SERTAD4 and immune cell infiltration in 33 cancer types (*p* > 0.05, ns. nonsignificant; *p* < 0.05 *; ).

The response to immunotherapy in CRC is closely associated with the gut microbiome,^30^ especially the complex relationship between immune cell infiltration within the TME and tumour treatment responses. Thus, we examined the relationship between SERTAD4 and 24 distinct subgroups of immune cells. Based on TCGA data, we found that only TH17 cells were negatively correlated with SERTAD4 (*R* = −0.167, *p*<0.001), and there was no correlation between NK CD56bright, NK CD56dim cells and SERTAD4 expression (*p* > 0.05), whereas the remaining 21 immune cells exhibited a positive correlation with SERTAD4 (Figure 6A–H and Figure S3A–P). Furthermore, we also examined the association between SERTAD4 expression and the infiltration of immune cells in other cancers (Figure 6I). These results strongly support the notion that SERTAD4 plays an immunologically relevant role in the TME of CRC.

**FIGURE 7:**
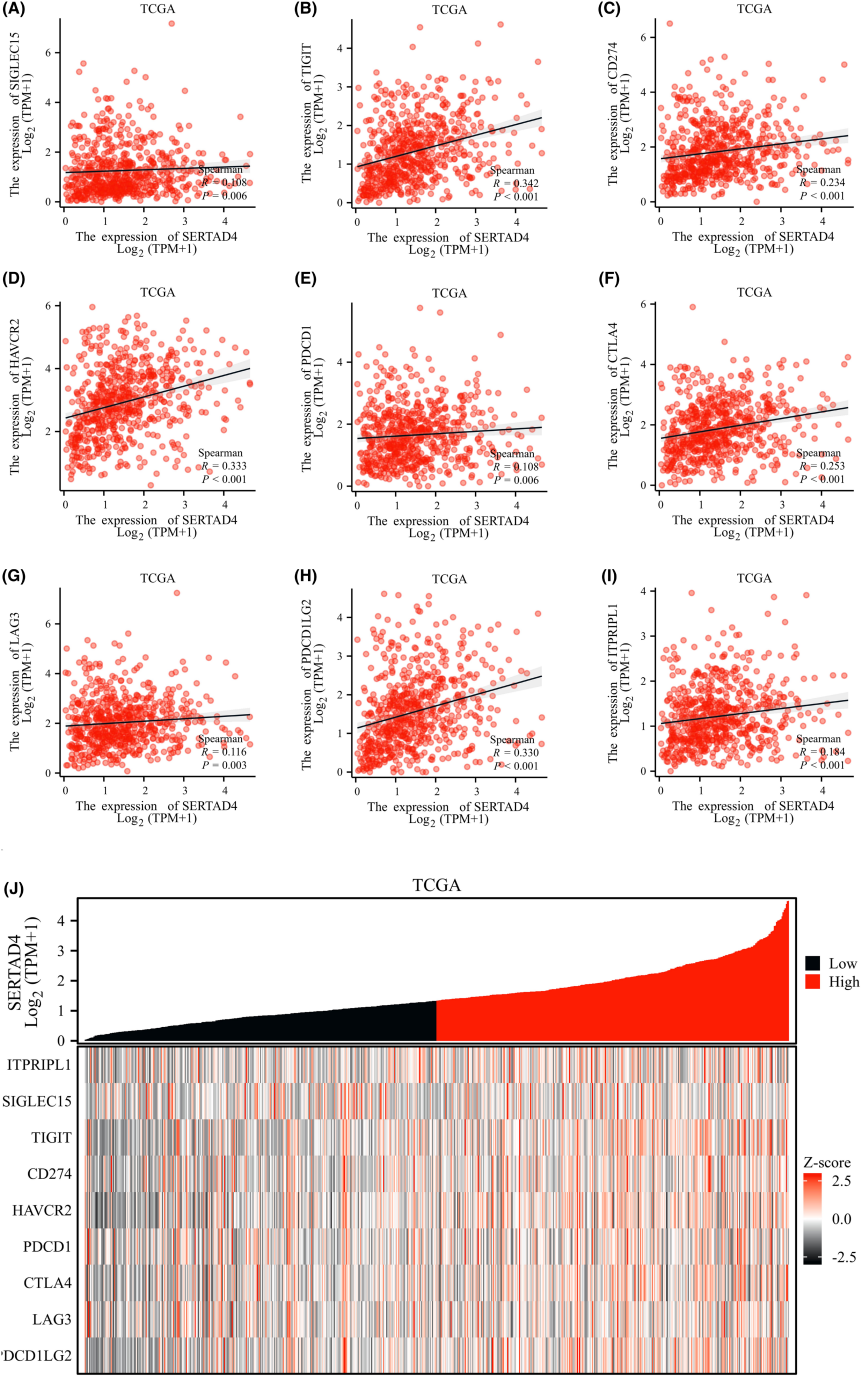
Correlation analysis between expression levels of SERTAD4 and immune checkpoint genes. The correlation of expression levels of SERTAD4 with SIGLEC15 (A), TIGIT (B), CD274 (C), HAVCR2 (D), PDCD1 (E), CTLA4 (F), LAG3 (G), PDCD1LG2 (H), and ITPRIPL1 (I). (J) Heatmap of co‐expression of SERTAD4 and immune checkpoint genes.

Connections between KRAS mutations and the response to immune checkpoint inhibitors in CRC treatment exist,^31^ and such connections may be influenced by the gut microbiome. Hence, we explored whether there is a correlation between immune checkpoint‐related genes and SERTAD4.^32^ We then retrieved the expression data for these eight genes from the TCGA database for more in‐depth analysis. The results showed that these representative immune checkpoint markers are showed a positive correlation with SERTAD4 (Figure 7A–I, all *p*<0.05). To further enhance the reliability of the study, we used a heatmap to display the relationship between these immune checkpoints and SERTAD4 (Figure 7J). These findings suggest that SERTAD4 may predict the effectiveness of immunotherapy in CRC patients.

### 3.4 Functional enrichment analysis of SERTAD4 in CRC

**FIGURE 8:**
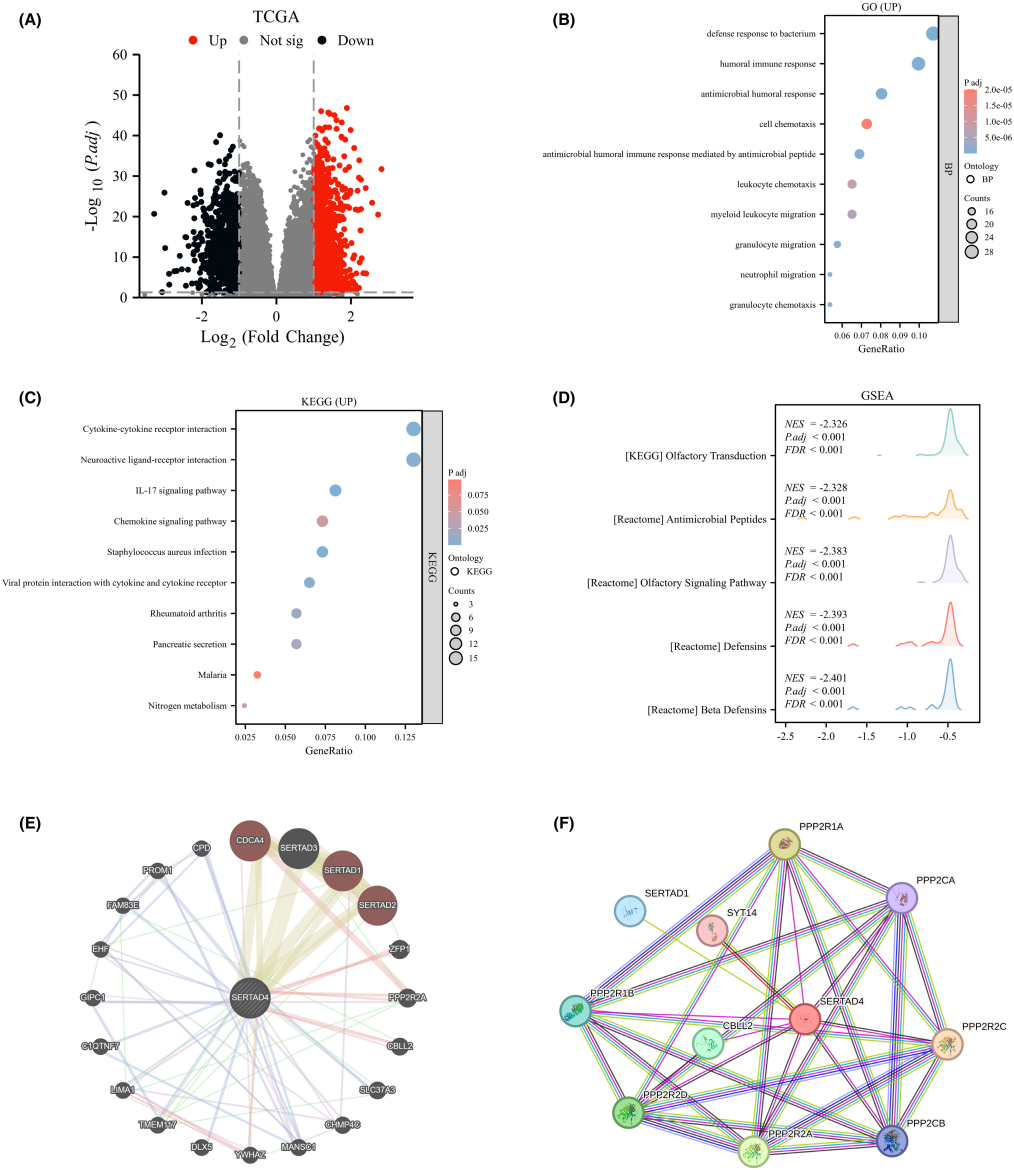
Pathway enrichment analysis of SERTAD4 in CRC. (A) Volcano plot of SERTAD4‐related DEGs. (B) GO analysis of BP activated by SERTAD4. (C) KEGG analysis of pathways activated by SERTAD4. (D) GSEA analysis of functions affected by expression differences of SERTAD4. (E) Gene interaction network diagram showing genes interacting with SERTAD4 analysed by Gene Mania. (F) Protein–protein interaction network (PPI) analysis of proteins interacting with SERTAD4.

Having established the differential expression of SERTAD4 in CRC and its importance in diagnosis, prognosis, and immunity, we proceeded to investigate the potential functions of SERTAD4. Based on TCGA data, we categorized the data into groups with high and low expression levels of SERTAD4 (to simulate overexpression or knockdown effects). We analysed the differential molecules between the two groups to infer the molecules possibly associated with SERTAD4. We detected 2163 significantly correlated DEGs caused by SERTAD4 (|fold change, FC| >1, *p* < 0.05) (Figure 8A). Given bioinformatics analyses and assay validation indicated that SERTAD4 is a protective factor in CRC, we were more interested in the activated pathways and functions of the DEGs. We found that the activated biological processes (BPs) included defence response to bacterium and so on (Figure 8B). In addition to BP, we also analysed the KEGG pathways and found that SERTAD4 activated Cytokine–cytokine receptor interaction, and so on (Figure 8C). These findings fully demonstrate that as a protective factor in CRC, SERTAD4 appears to exert anti‐cancer effects by enhancing antimicrobial capabilities and activating immune functions. This was further validated by GSEA analysis, supporting that SERTAD4 might function through ‘Antimicrobial Peptides’ and ‘Beta Defensins’ (Figure 8D).

To further explore genes that SERTAD4 may interact with, we used Gene MANIA (http://genemania.org) to analyse hundreds of datasets and billions of interactions from GEO, Bio GRID, IRefIndex, I2D and organism‐specific functional genomics datasets. Using Gene Mania, we constructed a gene–gene interaction network of SERTAD4 and changed neighbouring genes. The results showed that genes closely related to SERTAD4 included CDCA4, SERTAD3, SERTAD1 and SERTAD2, among others (Figure 8E). We further validated this through the STRING database, generating a protein–protein interaction (PPI) network for SERTAD4 (Figure 8F). We ultimately found that there are 32 edges and 11 nodes, including genes such as PPP2R2D, PPP2R2C, and PPP2R2A which are likely to interact with SERTAD4. Integrating both network diagrams, we identified PPP2R22 and CBLL2 as potential interactive proteins involved in anticancer role of SERTAD4, which will require further assay validation.

### 3.5 Validation SERTAD4 expression on the function of Fusobacterium nucleatum

**FIGURE 9:**
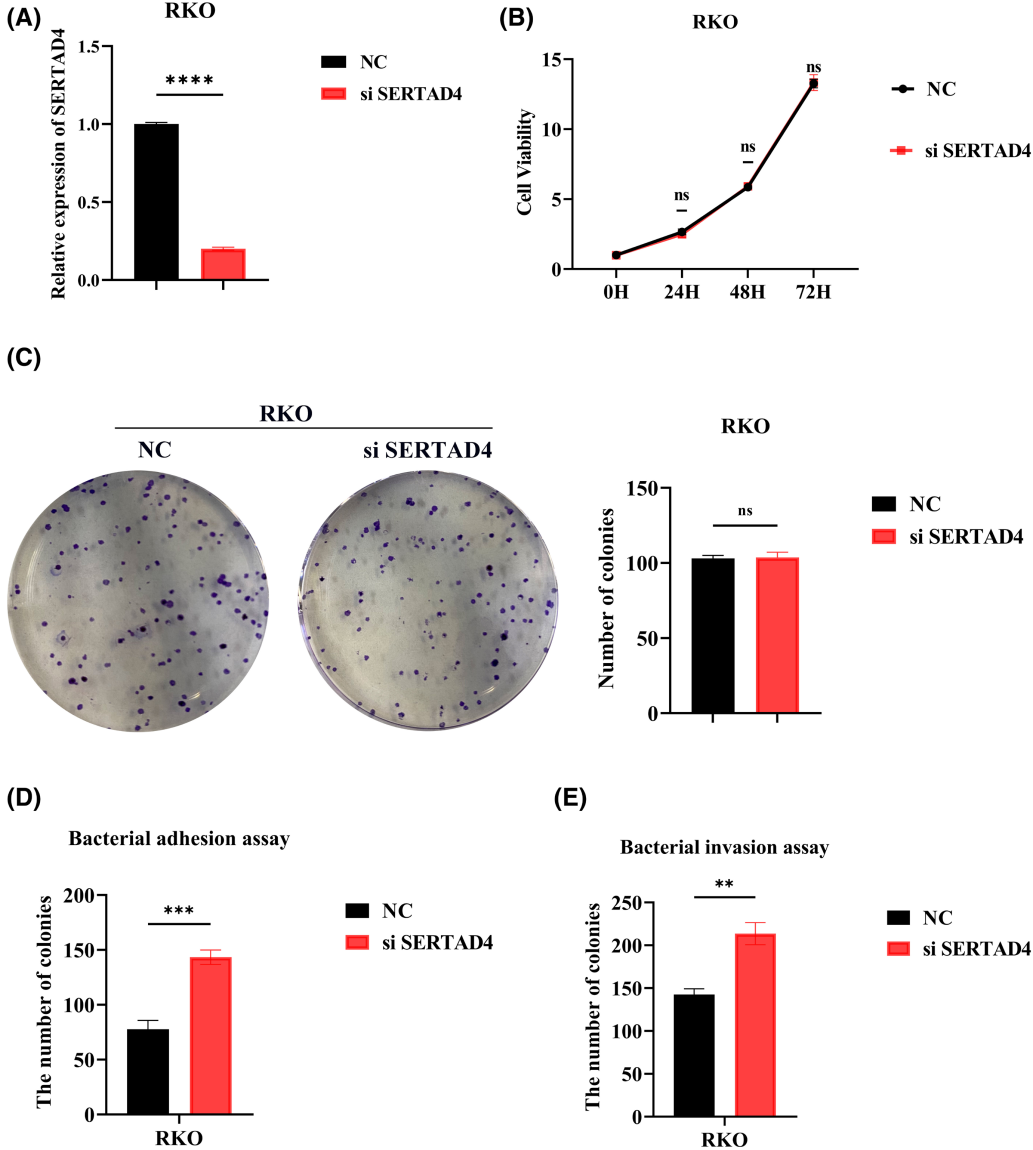
The impact of SERTAD4 expression on the function of FN. (A) RT‐qPCR confirmed the successful knockdown of SERTAD4. (B) CCK8 assay detected the effect of siSERTAD4 on proliferation of CRC. (C) Colony formation assay detected the effect of siSERTAD4 on stemness capability of CRC. (D) Bacterial adhesion assay detected the effect of siSERTAD4n on the adhesion ability of FN in CRC. (E) Bacterial invasion assay detected the effect of siSERTAD4 on invasion capability of FN in CRC. (*p* > 0.05, ns, nonsignificant; *p* < 0.01 **; *p* < 0.001 ***; *p* < 0.0001 ****; analyses were performed using Student's *t* test or Wilcoxon rank‐sum test, respectively).

Having identified the role of SERTAD4 in CRC, further assay validations are necessary. We first transfected RKO cells with siRNA and confirmed the successful knockdown of SERTAD4 via RT‐qPCR (Figure 9A). Through CCK8 assays, we evaluated whether SERTAD4 affects the proliferative capacity of CRC. The results indicated that the expression level of SERTAD4 does not impact CRC proliferation (Figure 9B,P >0.05). Furthermore, the colony formation also indicated SERTAD4 does not affect the stemness of CRC (Figure 9C,P >0.05). We conducted bacterial‐related phenotypic assays. The bacterial adhesion assay suggested that the suppression of SERTAD4 significantly enhances the adhesive ability of FN in CRC (Figure 9D,P <0.05). Additionally, the bacterial invasion assay also demonstrated that suppressing SERTAD4 significantly promotes the invasive ability of FN in CRC (Figure 9E,P <0.05). In summary, these results indicate that SERTAD4 functions as a tumour suppressor gene CRC by impeding the colonization of FN.

## 4 DISCUSSION

KRAS occupies a central position in the treatment of CRC. KRAS mutations activate limitless proliferation and survival of CRC cells, which is one of the key mechanisms in the development of CRC.^33^ Patients with KRAS mutation have a poor response to EGFR inhibitors,^34^ making it particularly important to test for KRAS gene status before formulating treatment plans. Multiple studies have shown that KRAS mutations have a close relationship with the prognosis of CRC patients. KRAS mutations tend to have worse outcomes and lower survival rates.^35^ Given the impact of KRAS mutations on treatment response and prognosis, developing molecular targets associated with KRAS mutations plays a significant role in personalized medicine. FN plays a complex and crucial role in CRC, with high abundance of FN promoting the development and progression of CRC.^36^, ^37^ Due to the unique anatomical location of CRC, the TME often engages in microbial exchanges with the gut microbiome. How KRAS mutations manipulate carcinogenic bacteria such as FN to alter the TME and, consequently, affect therapeutic efficacy and prognosis of CRC is a current research focus.

Using bioinformatics to analyse public databases for deep mining of potential diagnostic and prognostic biomarkers is an indispensable part of biological research, and this trend is increasingly growing.^38^, ^39^, ^40^, ^41^, ^42^ Single database bioinformatics cannot meet the rigour of research. Therefore, this study combined data from various databases for cross‐validation, ultimately further confirming results with patient samples and biological assays. Our study identified SERTAD4, the only gene associated with KRAS mutations and FN. SERTAD4 is a member of the SERTAD protein family.^43^ Previous research found that SERTAD4 establishes a dependent signalling pathway in the epigenetic reprogramming of heart failure.^44^ Additionally, SERTAD4 has been identified as a gene that affects cortical bone accumulation and bone strength.^45^ However, SERTAD4 has not been reported in any tumours, indicating that its specific functions and mechanisms in tumours present potential research opportunities.

This study is the first to reveal the protective role of SERTAD4 in CRC and its antitumorigenic function by inhibiting the colonization of FN. It also found correlations between SERTAD4 and various immune cell infiltrates. An intriguing phenomenon uncovered by our research is that SERTAD4 expression is not only reduced in CRC tissues but is also suppressed following KRAS mutations and FN infection. In these conditions, the expression of SERTAD4 may indicate its important role in maintaining normal functions of intestinal epithelial cells, and its reduction could facilitate the growth and survival of tumour cells and carcinogenic bacteria. Correspondingly, cross‐validation using TCGA database and data from Fujian Provincial Hospital patients revealed that lower levels of SERTAD4 suggest a worse survival rate of CRC patients. Thus, the expression of SERTAD4 could act as a biomarker for predicting the clinical outcome of CRC patients.

CEA is widely used in the diagnosis and monitoring of treatment efficacy in CRC.^46^ Through cross‐validation with multiple independent datasets, we found that SERTAD4 is a reliable diagnostic target of CRC, with diagnostic efficacy comparable to that of CEA. Future research should focus more on the combined application of multiple targets for the diagnosis of early‐stage CRC. The combination of CEA and SERTAD4 holds promise as a strategy for diagnosing and screening CRC patients.

The TME is a key factor in tumour development, encompassing tumour cells, immune cells, extracellular matrix and intratumoral microbes.^47^ The TME is considered a potential target of cancer treatment.^48^ In the TME, various immune cells interact with tumour. These immune cells could either inhibit tumour cells or be manipulated by tumour cells to promote tumour growth and metastasis.^49^, ^50^ Previous findings have confirmed the protective role of SERTAD4 in CRC. Additionally, analyses disclosed a correlation between elevated levels of SERTAD4 expression and increased immune cell infiltration. Notably, high levels of SERTAD4 inhibited TH17 cell infiltration, which may relate to the unique role of TH17 cells in CRC. Studies have found that Th17 cells trigger epithelial‐mesenchymal transition to promote CRC metastasis.^51^ And Th17 cells can also synergistically activate STAT3 and NF‐kB to promote CRC.^52^ The extent and type of immune cell infiltration in the TME are significant for tumour prognosis and treatment outcome. Moreover, FN can regulate immune cell composition and function in the TME by promoting polarization of tumour‐associated macrophages, reducing cytotoxic T cells.^53^, ^54^, ^55^ These regulations influence tumour immune escape and prognosis. Hence, the enrichment of immune cell infiltration by SERTAD4 in the TME is crucial for CRC. In summary, there is a complex interplay between immune cell infiltration within the TME and FN. SERTAD4 plays a crucial role in regulating these interactions, which significantly impacts tumour progression, tumour immune escape mechanisms and the efficacy of immunotherapy. Future research needs to delve deeper into the specific mechanisms of these interactions to develop more effective tumour treatment methods.

Immune checkpoint‐related genes are part of the immune system that cancer cells exploit to evade immune elimination. High expression of immune checkpoint genes often signifies tumour cell immune escape, further promoting tumour progression.^56^, ^57^ Surprisingly, in our study, SERTAD4, a protective factor in CRC, showed positive correlation with immune checkpoint molecules. This may be because immune checkpoint‐related genes may not only play roles in immune escape and suppressing tumour immune response but might also be involved in activating and regulating positive immune responses, especially under specific cell types and microenvironment conditions. Considering the complexity of the TME, the upregulation of immune checkpoint genes could be a result of FN stimulation in the TME. This positive correlation might also reveal the complex signalling network between tumour cells and immune cells. SERTAD4 possibly serve as a key node in these networks, functioning through interactions with immune checkpoint‐related genes. Overall, this phenomenon warrants further in‐depth research, which could provide significant insights for developing new treatment strategies and improving patient prognosis.

The bacterial adhesion and invasion abilities are new, critical areas of CRC research.^58^ The attachment of FN to intestinal epithelial cells disrupts normal cell function, induces inflammation and is a key factor in tumour development.^7^, ^36^ Further invasion directly harms intestinal cells, creating favourable conditions for tumour occurrence and development. Future research should focus on inhibiting bacterial adhesion and invasion abilities on CRC cells. After bioinformatics analysis and tumour patient sample revealed potential protective role of SERTAD4 in CRC. Bacterial phenotype assays confirmed that SERTAD4 directly inhibits adhesion and invasion abilities of FN. And SERTAD4 could not affect CRC proliferation and stemness capabilities. Interestingly, FN can suppress SERTAD4 expression, and a lower level of SERTAD4 also loses the ability to inhibit the colonization of FN in CRC. This phenomenon further underscores the significance of SERTAD4 in controlling FN, warranting further investigative efforts into the mechanisms. Understanding the molecular mechanisms of bacterial adhesion and invasion in CRC is not only significant for comprehending tumour genesis but could also provide targets for developing new CRC treatments, such as antimicrobial treatments or methods to adjust gut microbiome balance.

In this study, we conducted a systematic and comprehensive analysis and validation of SERTAD4 in CRC. However, some limitations should be considered. First, although we cross‐validated with GEO datasets, some key GEO datasets may have been omitted. Secondly, the exact molecular mechanisms by which SERTAD4 inhibits colonization of FN in CRC remain unclear.

## 5 CONCLUSION

In conclusion, this study reveals the important role of SERTAD4 in CRC and provides significant insights for understanding the molecular mechanisms of CRC and developing new therapeutic strategies. As a molecular marker associated with KRAS mutations and FN infection, SERTAD4 is abnormally reduced in CRC. SERTAD4 is related to immunotherapy and influences CRC progression by affecting the colonization of FN in CRC. SERTAD4 can serve as a new biomarker for the diagnosis, prognosis and prediction of immunotherapy efficacy in CRC patients. Future research should delve further into the specific functions and mechanisms of action of SERTAD4, as well as its potential applications in CRC treatment.

## AUTHOR CONTRIBUTIONS

**Yizhen Chen:** Data curation (equal); methodology (equal); software (equal); supervision (equal); validation (equal); visualization (equal); writing – original draft (equal); writing – review and editing (equal). **Yuanyuan Zheng:** Supervision (equal); validation (equal); visualization (equal); writing – original draft (equal); writing – review and editing (equal). **Shaolin Liu:** Validation (equal); visualization (equal).

## FUNDING INFORMATION

This research was supported by the Joint Funds for innovation of science and technology of Fujian Province (Project no.: 2023Y9290); the Startup Fund for Scientific Research, Fujian Medical University from Yizhen Chen (Project no.: 2023QH2040).

## CONFLICT OF INTEREST STATEMENT

The authors have no conflict of interest. All authors had full access to all of the data in the study and had final responsibility for the decision to submit for publication.

## Supporting information


Data S1.

## Data Availability

Data for this study may be requested from the corresponding author where appropriate.
