# Supplementary material for: KRAS mutation promotes the colonization of Fusobacterium nucleatum in colorectal cancer by down‐regulating SERTAD4
Source: J Cell Mol Med. 2024 Oct 27;28(20):e70182. doi: 10.1111/jcmm.70182 (PMC11512757; doi:10.1111/jcmm.70182)
Supplement: Supplementary file 1 — Data S1. [file JCMM-28-e70182-s001.docx]

**Figure S1. RNA levels of SERTAD4 in cancer**


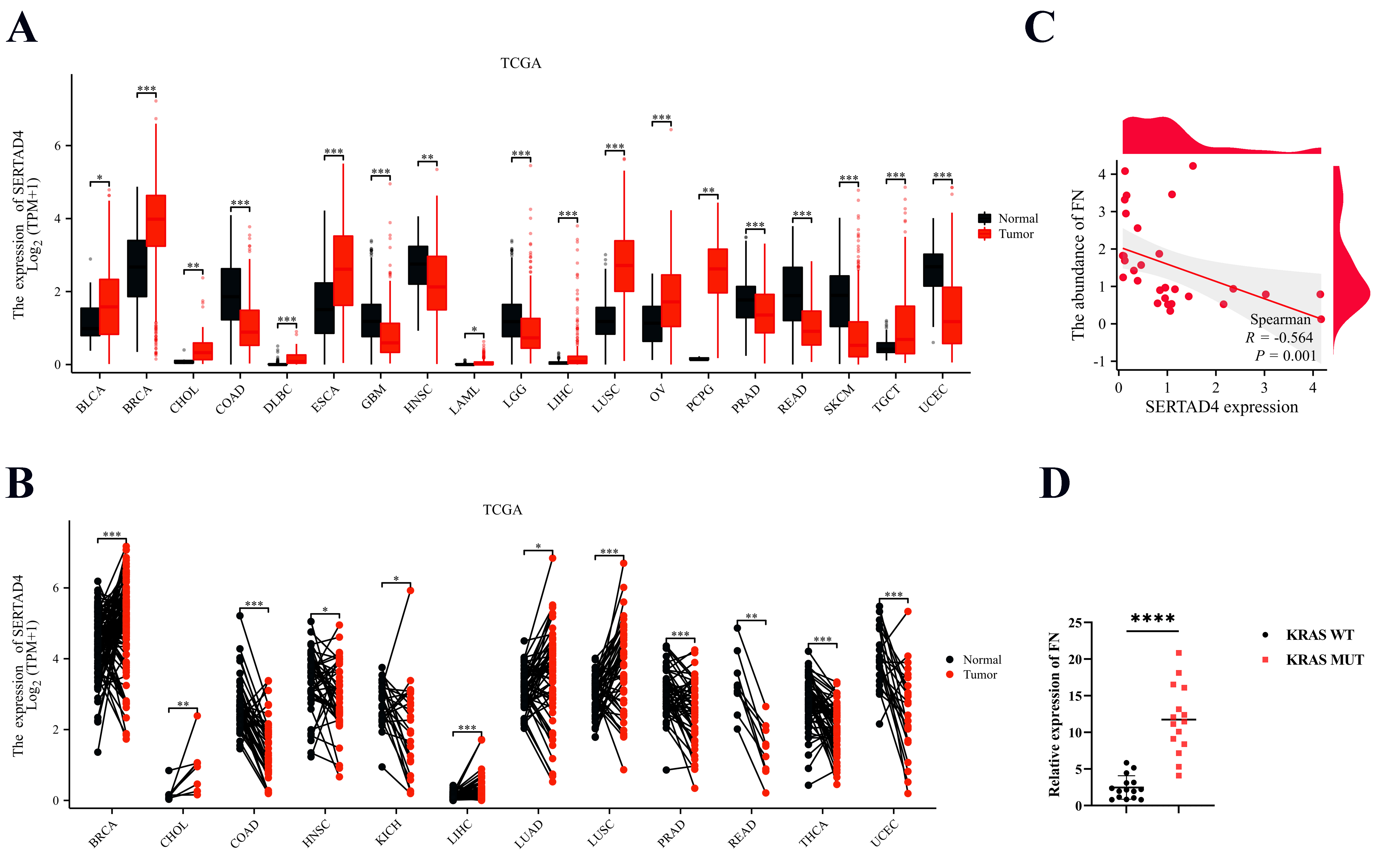


(A) Based on TCGA, the RNA levels of SERTAD4 in 19 cancer tissues and normal tissues were detected. (B) Based on TCGA, the RNA levels of SERTAD4 in 12 cancer tissues and matched normal tissues were detected. (C) RT-qPCR was used to detect the correlation between SERTAD4 expression and the abundance of FN in 30 CRC tissues. (D) qPCR was used to detect the correlation between KRAS mutation and the abundance of FN in 30 CRC tissues. (P＞0.05, ns. nonsignificant; P < 0.05 *; P < 0.01 **; P < 0.001 ***; P < 0.0001 ****; analyses were performed using Student’s t test or Wilcoxon rank-sum test, respectively).

**Figure S2. Low expression of SERTAD4 indicates poor prognosis in CRC patients**


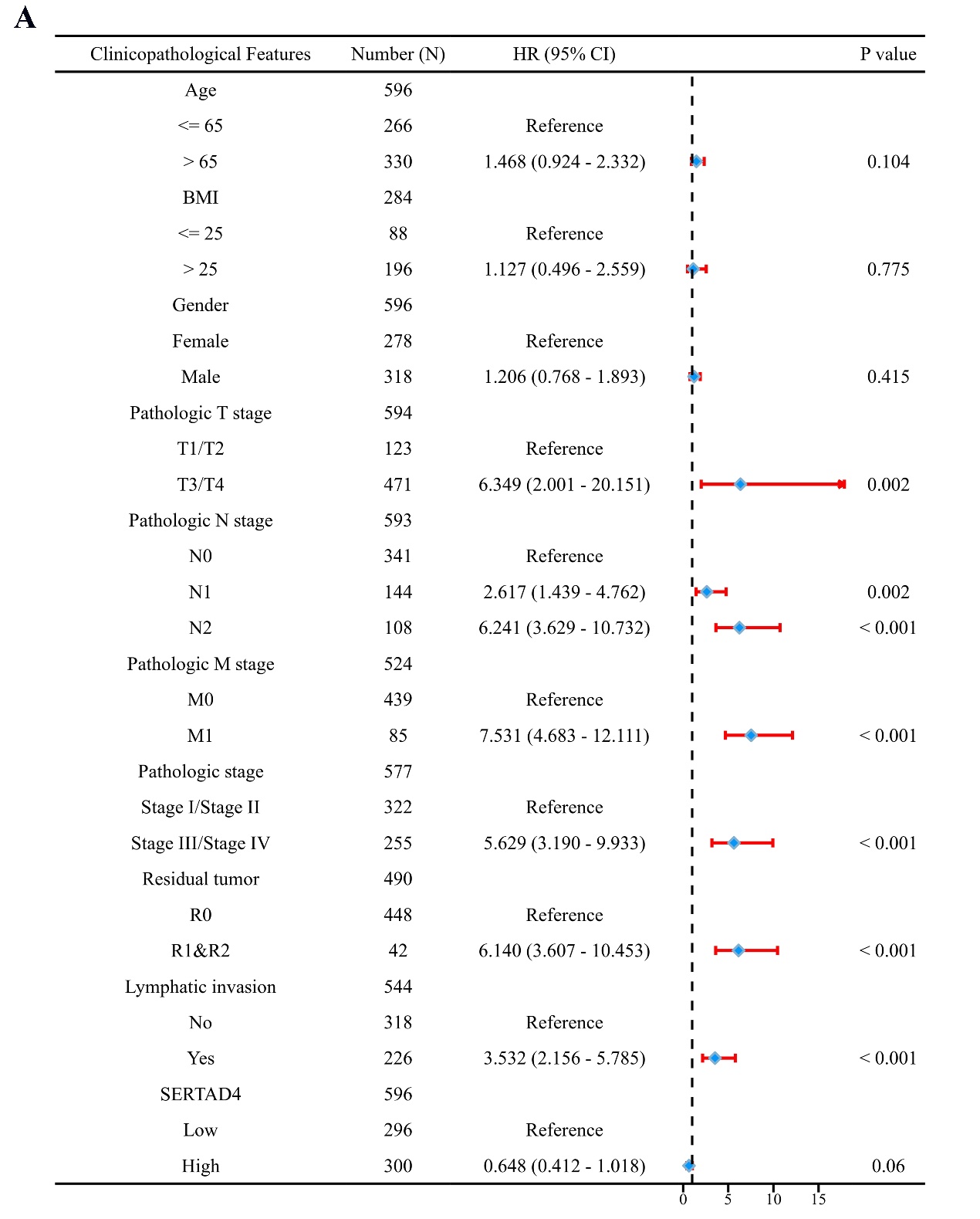


(A) A forest plot showing cox regression analysis of the impact of SERTAD4 expression on DSS in CRC (P＞0.05, ns. nonsignificant; P < 0.05 *; P < 0.01 **; P < 0.001 ***; P < 0.0001 ****; analyses were performed using Student’s t test or Wilcoxon rank-sum test, respectively).

**Figure S3. The relationship between expression levels of SERTAD4 and immune cell infiltration**


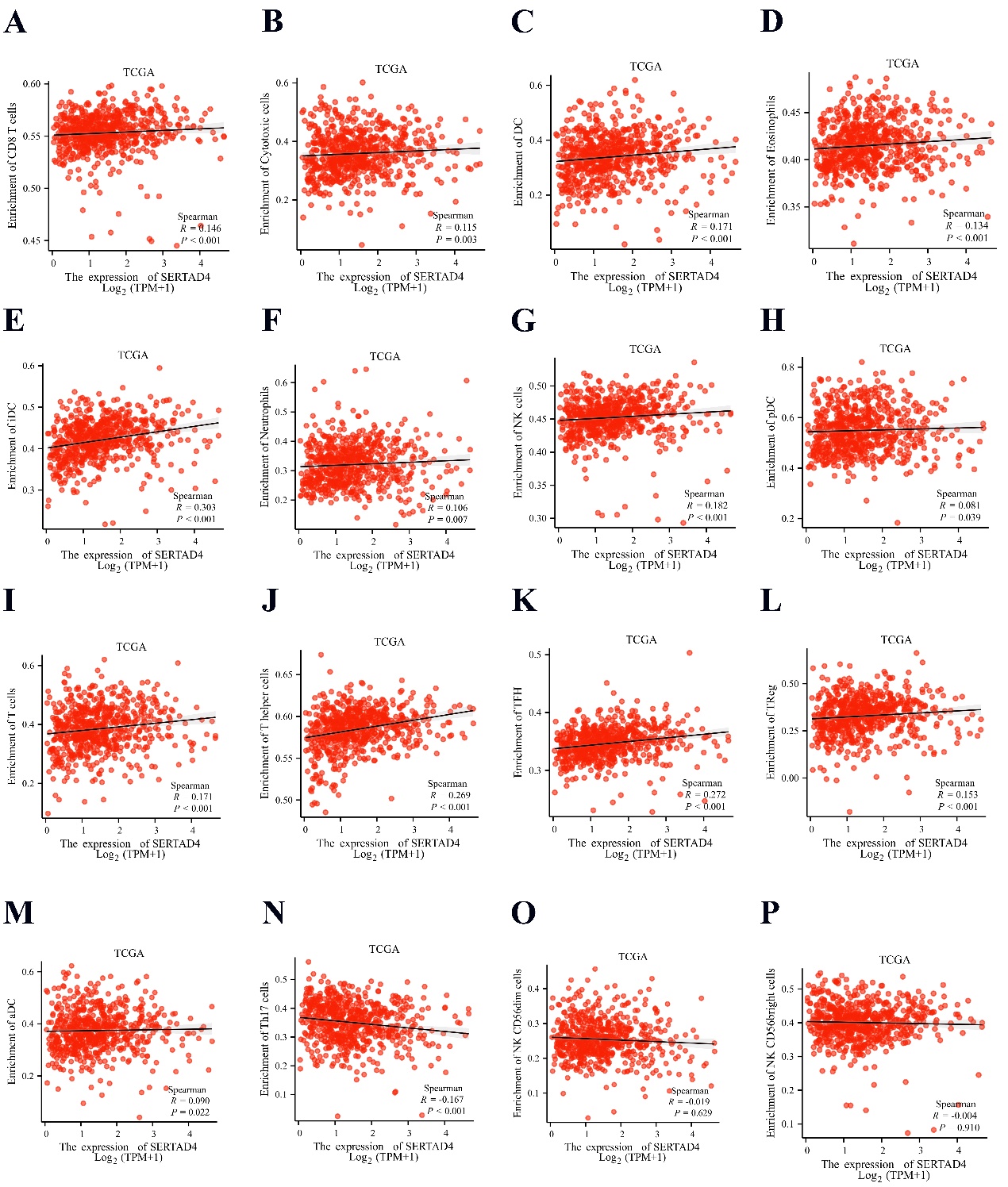


The correlation of expression levels of SERTAD4 with cd8t (A), cytotoxic cell (B), dc (C), eosinophils (D), idc (E), neutrophils (F), nk cell (G), pdc (H), t cell (I), t helper (J), tfh (K), treg (L), adc (M), th17 cell (N), cd56dim cell (O), cd56bright cell (P). (P＞0.05, ns. nonsignificant; P < 0.05 *; P < 0.01 **; P < 0.001 ***; P < 0.0001 ****; analyses were performed using Student’s t test or Wilcoxon rank-sum test, respectively).

**Table S1 Clinic-pathological characteristics of CRC patients**

| Case Number | Gender | Age | TNM stage | KRAS staus |
| --- | --- | --- | --- | --- |
| 1 | Female | 52 | I | G12D |
| 2 | Female | 58 | III | G12V |
| 3 | Female | 65 | II | G12D |
| 4 | Female | 58 | I | G12D |
| 5 | Male | 69 | II | G12C |
| 6 | Male | 71 | II | G12D |
| 7 | Female | 50 | II | G12V |
| 8 | Male | 56 | II | G12D |
| 9 | Male | 55 | II | G13D |
| 10 | Male | 59 | III | G12V |
| 11 | Male | 75 | II | G13D |
| 12 | Male | 73 | II | G12D |
| 13 | Male | 52 | II | G12V |
| 14 | Female | 54 | II | G13D |
| 15 | Male | 64 | III | G12D |
| 16 | Male | 60 | II | WT |
| 17 | Male | 49 | II | WT |
| 18 | Male | 59 | I | WT |
| 19 | Male | 65 | II | WT |
| 20 | Female | 62 | II | WT |
| 21 | Female | 55 | I | WT |
| 22 | Female | 49 | II | WT |
| 23 | Female | 66 | III | WT |
| 24 | Male | 67 | II | WT |
| 25 | Male | 73 | II | WT |
| 26 | Male | 62 | I | WT |
| 27 | Male | 70 | II | WT |
| 28 | Male | 71 | II | WT |
| 29 | Male | 64 | II | WT |
| 30 | Female | 69 | III | WT |

WT: wild-type.
